# How Do University Students’ Perceptions of the Instructor’s Role Influence Their Learning Outcomes and Satisfaction in Cloud-Based Virtual Classrooms During the COVID-19 Pandemic?

**DOI:** 10.3389/fpsyg.2021.627443

**Published:** 2021-04-16

**Authors:** Rong Wang, Jiying Han, Chuanyong Liu, Hongji Xu

**Affiliations:** ^1^Department of Physiology and Pathophysiology, School of Basic Medical Sciences, Shandong University, Jinan, China; ^2^School of Foreign Languages and Literature, Shandong University, Jinan, China; ^3^School of Information Science and Engineering, Shandong University, Qingdao, China

**Keywords:** instructors’ role, learning outcomes, learning satisfaction, academic self-efficacy, cloud-based virtual classrooms

## Abstract

This study examined the relationships between the role of the instructor and university students’ learning outcomes in cloud-based classrooms during the COVID-19 (coronavirus disease 2019) pandemic. The results of an online survey of 7,210 university students in mainland China revealed that the students’ perceived learning outcomes and learning satisfaction were positively related to instructor innovation and negatively related to instructor performance. Instructional support was positively related to the students’ perceived learning outcomes but not directly related to their learning satisfaction. The students’ academic self-efficacy mediated the influence of instructional support and instructor innovation on their perceived learning outcomes and learning satisfaction. The results contribute to knowledge of the role instructors play in facilitating students’ learning outcomes in higher education and suggest ways to improve the learning environment and learning outcomes, especially in cloud-based virtual classrooms.

## Introduction

The learning environment, whether traditional or cloud-based, is a significant determinant of students’ learning process and outcomes (Eom et al., 2016; Al-Samarraie and Saeed, 2018). In the last few decades, the role of instructors in shaping students’ learning experience and learning outcomes has been widely acknowledged (Martin et al., 2019). With increasing attention being paid to online learning environments, instructors are now regarded as “facilitators” (Martin et al., 2018) of student learning. However, most studies on online learning have been conducted in either asynchronous online settings or blended environments (Shea et al., 2006; Hew, 2015). Very little is known about the role instructors play in synchronous learning environments (Kuo et al., 2014a; Martin et al., 2018), wherein the availability of cloud services has greatly improved the efficiency of communication between students and instructors (Baanqud et al., 2020).

The sudden outbreak of COVID-19 (coronavirus disease 2019) in mainland China and the subsequent pandemic resulted in the cancelation of all face-to-face teaching activity at all levels of education throughout the country. In higher education, under the guidance of China’s Ministry of Education, cloud-based e-learning has served as a substitute for the traditional classroom or the blended learning environment since late February 2020. This reflects a change from forced online learning to the “new normal” e-learning environment (Parmigiani et al., 2019; Pham and Ho, 2020). Within this changed learning environment, how do university students perceive their learning outcomes and their instructors’ performance? How do instructors influence their students’ learning? This study aimed to address these questions by exploring the influence of instructors on undergraduate students’ learning in the cloud-based virtual classroom in mainland China.

## Literature Review

### The Role of Instructors in the Online Learning Environment

Research on learning environments has been expanding for about five decades. According to an extensive literature review, research in this field has generated considerable insights into the relationships between aspects of the learning environment and earning outcomes (Allen and Fraser, 2007). For example, research has shown that, in the online learning environment, instructors’ use of cloud-supported collaborative tools is positively related to the development of learners’ knowledge construction skills (Baanqud et al., 2020). The relationships between the learning environment and students’ cognitive and affective outcomes, such as motivation, efficacy, and engagement (Allen and Fraser, 2007; Alqurashi, 2016; Xu et al., 2020), have become one of the most important lines of inquiry in studies of the learning environment. The results of these studies have provided convincing evidence that the role of instructors is a significant determinant or predictor of student learning outcomes in both traditional and online learning settings (Eom et al., 2016; Martin et al., 2018; Yunusa and Umar, 2021).

A favorable learning environment improves students’ learning outcomes (Kember et al., 2010). In the online learning environment, the instructor’s role is one of the most important factors contributing to effective online teaching (Eom et al., 2016; Martin et al., 2019), in addition to commonly identified environmental factors such as the teaching technology, the course content, student–teacher interaction, and the learning model (Piccoli et al., 2001). Studies have indicated that in online learning environments, instructors can enhance students’ understanding of the course content, acknowledge student engagement, and motivate students to explore new knowledge through various facilitation strategies and cloud computing tools (Martin et al., 2018, 2019; Xu et al., 2020). Instructors can also improve students’ learning outcomes and learning satisfaction by using multiple scaffolding strategies in online environments (Eom et al., 2016; Mamun et al., 2020).

Our review of studies exploring the role instructors play in student learning revealed that this topic has mainly been assessed in relation to instructional support (Kuo et al., 2014b), instructor performance (Ali and Ahmad, 2011), and instructor innovation (Lee, 2011). Students’ perceptions of the quality of differentiated support for learning are one of the most important components influencing their independent learning and motivation (Lee et al., 2011; Mamun et al., 2020). In online learning environments, perceived instructional support includes timely and constructive feedback, clear instructions and explanations, and various scaffolding strategies from instructors (Lee et al., 2011; Martin et al., 2018; Mamun et al., 2020). Instructional support is a critical predictor of students’ satisfaction and perceived learning outcomes in asynchronous online learning settings (Mullen and Tallent-Runnels, 2006; Yunusa and Umar, 2021).

Instructor innovation is the extent to which an instructor plans new and unusual class activities, teaching techniques, and assignments to establish a flexible learning environment that nevertheless maintains orderly and clear expectations (Fraser and Treagust, 1986). As a solution to challenges faced in contemporary education and as a mediator for change and adaption, instructor innovation improves students’ learning outcomes and satisfaction (Walder, 2017). Current knowledge of the influence of instructor innovation on university student learning outcomes remains very limited in reference to online learning environments, although a preliminary study conducted in an asynchronous online environment suggested that instructor innovation is positively related to student learning satisfaction (Lee, 2011). The literature has also indicated that the application of appropriate e-learning strategies and skills to online teaching helps to improve teaching effectiveness and students’ engagement and motivation (Xu et al., 2020).

Instructors play a vital role in creating successful online learning environments (Ali and Ahmad, 2011). They need to continuously acquire new skills and expertise to facilitate students’ learning process and improve their learning outcomes (Martin et al., 2018). A good instructor must be able to ensure the expected interactions at the instructor–learner, learner–learner, and learner–content/technology levels (Beaudoin, 2009). They must also be able to determine appropriate learning tasks and tests based on the differences between individual students (Banerjee and Brinckerhoff, 2002). Similarly, the instructor’s attitude toward and control of technology are a critical determinant of the success of e-learning and the effectiveness of students’ perceived e-learning environments (Selim, 2007).

### Students’ Perceived Learning Outcomes and Learning Satisfaction

Across various learning settings, the literature has widely acknowledged that a proper learning environment can improve learning outcomes either directly or indirectly (Chang and Fisher, 2001; Allen and Fraser, 2007). To date, a number of variables have been used as indicators of students’ multiple learning outcomes in educational research, such as examination scores (Hamer, 2000), task performance and goal achievement (Deeter-Schmelz et al., 2002), and students’ perceived learning outcomes and satisfaction (Eom et al., 2016). Students’ perceived learning outcomes and learning satisfaction have been widely used as indicators of the quality of online learning (Eom et al., 2016). Students’ perceptions of learning outcomes, which are based on how well students believe they have done in a course, provide important insights to inform course development (Rundle-Thiele, 2005; Kuhn and Rundle-Thiele, 2009). Students’ learning satisfaction, defined as a short-term attitude resulting from students’ evaluation of their educational experience, services, and facilities (Mukhtar et al., 2015), has been measured in terms of students’ perceptions of the quality of learning in an online course, their enjoyment of the online course, and whether they would recommend the course to other students (Lee et al., 2011).

Many studies exploring the relationship between the learning environment and learning outcomes have indicated that instructors, a key predictor of satisfaction and perceived learning, help to improve student learning outcomes or satisfaction in online learning settings (Martin et al., 2018; Yunusa and Umar, 2021). Instructors have been found to be capable of motivating students to learn through instructional support (Mullen and Tallent-Runnels, 2006) and instructor innovation (Lee, 2011). However, as these studies have been conducted mainly in asynchronous online settings, very little is known about the influence of instructors, especially in terms of instructional support, instructor innovation, and instructor performance, on students’ perceived learning outcomes and satisfaction in cloud-based virtual classrooms, a purely synchronous online learning setting.

### Students’ Academic Self-Efficacy as a Mediator

Self-efficacy in the traditional learning environment, also known as academic self-efficacy (ASE), has been widely defined as one’s belief in one’s confidence and ability to successfully accomplish a specific learning task in an educational setting (Bandura, 1986). This measure reflects students’ expectations of how successful they will be in the classroom (Bandura, 1997). In addition to research examining the four sources of self-efficacy, i.e., enactive mastery experience, vicarious experience, verbal persuasion, and physiological and affective states (Bandura, 1997), several studies have investigated the influence of situational and instructional factors on students’ self-efficacy in online learning environments, especially in higher education (Fryer and Bovee, 2016). Other studies have demonstrated that instructors help to foster students’ self-efficacy through support connected to the above four sources of self-efficacy (Abbitt and Klett, 2004). In addition, research has consistently demonstrated that self-efficacy, a key element of social cognitive theory, is a significant contributor to students’ learning outcomes (e.g., Schunk and Pajares, 2009).

Regarding the online learning environment, researchers have identified computer self-efficacy (CSE) and Internet self-efficacy (ISE) as significant variables in addition to ASE. “CSE” refers to an individual’s perceived confidence regarding their ability to use a computer and has been found to be related to students’ learning outcomes in the computer-based learning environment (Moos and Azevedo, 2009). “ISE” refers to the self-assessment of one’s ability to perform Internet technology-related activities to produce desirable results. With a prevailing interest in ISE rather than ASE in online learning environments, studies have demonstrated that students’ ISE is positively related to their learning satisfaction and learning outcomes (Kuo et al., 2014b). However, most relevant studies have been conducted in asynchronous or blended online learning environments. A recent study conducted in a synchronous online learning environment revealed that students’ ISE did not significantly contribute to their learning satisfaction, as the synchronous learning system may not require significant skills to perform Internet-related tasks (Kuo et al., 2014a). The positive influence of ASE on students’ learning outcomes and learning satisfaction has been extensively researched, and the results have indicated that ASE may be a causal, mediating, or moderating factor explaining the relationship between ASE and the academic performance of university students (Honicke and Broadbent, 2016). However, Yukselturk and Bulut (2007) found that ASE did not predict students’ Internet-based learning success. Thus, the relationship between ASE and students’ success in online learning environments remains debatable (Tsai et al., 2011).

Based on the aforementioned literature, this study aimed to investigate the relationships between undergraduate students’ perceptions of the role of their instructors and their own ASE and learning outcomes, focusing on cloud-based virtual classrooms in universities in mainland China. Specifically, the study was designed to address the following questions: (1) What are the relationships between students’ perceptions of the instructor’s role and students’ perceived ASE, learning outcomes, and learning satisfaction? (2) Does ASE mediate the relationships between students’ perceptions of the instructor’s role and their own learning outcomes and learning satisfaction?

## Methodology

### Participants

In April 2020, an online questionnaire was administered to university students in a province in eastern China. The sample comprised 7,210 undergraduate students (62.2% female, 37.8% male) from two universities: a national research-oriented university (*n* = 5,405, 70.0%) and a provincial teaching-oriented university (*n* = 2,165, 30.0%). In terms of their year of study, 53.2% of the participants were freshmen, 24.7% were sophomores, 17.6% were juniors, and 4.5% were seniors. In terms of academic discipline, the sample comprised students majoring in the social sciences and humanities (55.8%), science and technology (41.2%), and medicine (3.0%).

### Instruments

The online questionnaire had two parts and comprised 32 items. The first part collected demographic information such as gender, grade, major, and type of university. The second part consisted of four measures assessing the students’ perceptions of the role of online instructors and their own online ASE, learning outcomes, and learning satisfaction. The items were slightly modified to indicate the online learning environment. All were scored on a 5-point Likert-type scale ranging from 1, “strongly disagree,” to 5, “strongly agree” (Table 1).

**TABLE 1:** Structural equation modeling testing the factor loading, reliability, measurement error, composite reliability, and AVE (*n* = 7,210).

Construct, CFA fit	Factor (no. of items)	Item	Factor loading	Square multiple correlation (SMC)	Measurement error	Cronbach α	Composite reliability (CR)	Average variance extracted (AVE)
The role of instructors (χ^2^ = 7761.83, *df* = 116, *p* < 0.001, CFI = 0.92, TLI = 0.91, RMSEA = 0.069)	Instructor performance (7)	The instructors were available for online consultation during office hours	0.74	0.55	0.34	0.93	0.93	0.67
		The instructors stimulated student learning	0.79	0.62	0.23			
		The instructors treated all students fairly	0.80	0.64	0.21			
		The instructor treated all students with respect	0.87	0.75	0.14			
		The instructor welcomed and encouraged questions and comments	0.86	0.74	0.14			
		The instructor presented the information clearly	0.85	0.72	0.15			
		The instructor emphasized the major points and concepts	0.82	0.67	0.19			
	Instructional support (6)	The course goals/objectives were clearly outlined	0.77	0.59	0.31	0.90	0.91	0.62
		I knew what I was expected to accomplish each week	0.75	0.57	0.21			
		The instructors provided clear instructions for assignments and quizzes	0.69	0.51	0.47			
		The courses provided relevant resources	0.83	0.69	0.20			
		The feedback on the assignments was helpful	0.81	0.66	0.21			
		I felt that I could ask any questions regarding the course materials to the instructors	0.77	0.59	0.24			
	Instructor innovation (4)	The instructors adopted different teaching methods from those in face-to-face courses	0.78	0.61	0.26	0.91	0.93	0.76
		The instructors designed new online learning activities to get us involved	0.87	0.76	0.28			
		The instructors used various and innovative teaching methods in the online courses	0.90	0.81	0.18			
		The instructors assigned different learning tasks in online courses	0.93	0.68	0.15			
Academic self-efficacy (4) (χ^2^ = 39.66, *df* = 2, *p* < 0.001, CFI = 0.99, TLI = 0.99, RMSEA = 0.073)		When compared to other students, I am certain that I can do well on the lesson assignments	0.83	0.69	0.28	0.88	0.90	0.76
		I believe that I can understand the concepts taught in online courses	0.88	0.78	0.18			
		I can utilize effective study skills in learning new concepts in online courses	0.90	0.81	0.15			
Students’ perceived outcomes (3) (χ^2^ = 141.44, *df* = 2, *p* < 0.001, CFI = 0.99, TLI = 0.99, RMSEA = 0.073)		I feel that online learning improved my learning performance	0.92	0.85	0.16	0.95	0.95	0.87
		I feel that online learning enhanced my effectiveness for learning	0.95	0.89	0.12			
		I found online learning useful		0.93	0.86	0.15		
Students’ learning satisfaction (5) (χ^2^ = 115.74, *df* = 5, *p* < 0.001, CFI = 0.97, TLI = 0.98, RMSEA = 0.069)		The courses increased my interests in the subject	0.86	0.75	0.29	0.93	0.94	0.74
		I felt I achieved the objectives in online courses	0.75	0.56	0.38			
		I liked the online course format	0.87	0.76	0.32			
		I felt comfortable in online courses	0.89	0.79	0.23			
		I would recommend the courses to others	0.93	0.86	0.17			

#### The Role of Instructors

Students’ perceptions of the instructor’s role were assessed in three dimensions: instructional support, instructor innovation, and instructor performance. Students’ perceptions of instructional support were assessed using six items proposed by Lee et al. (2011), with slight adaptations to indicate the online learning environment. Instructor innovation was measured using four items adapted from the College and University Classroom Environment Inventory (Fraser et al., 1986; Johnson et al., 2000). Seven additional items were selected from Tallent-Runnels et al. (2005) to assess instructor performance.

#### Academic Self-Efficacy

To assess students’ ASE in online learning, four items representing general ASE were adapted from the Learning Motivation Questionnaire (Lim and Kim, 2003). The adapted translation of these items was based on Bandura’s (2006) suggestion that items measuring self-efficacy should be phrased in terms of “can do” rather than “will do.”

#### Students’ Perceived Learning Outcomes

Students’ perceptions of the outcomes of online learning were assessed using three items adapted from the study of Eom et al. (2016).

#### Learning Satisfaction

Students’ learning satisfaction was assessed by five items adapted from Lee et al. (2011).

### Data Analysis

To determine whether the measures had satisfactory psychometric properties, exploratory factor analysis (EFA) using SPSS 22.0 software was first conducted to determine the factor structure. Next, confirmatory factor analysis (CFA) was conducted using AMOS 22.0 software to evaluate the item reliability, convergent validity, and discriminant validity. The reliability of the subscales was assessed by Cronbach α coefficients using SPSS 22.0. Repeated-measures one-way analysis of variance was conducted to evaluate whether there was a significant difference between the mean scores for the students’ perceptions of the instructor’s role, ASE, learning outcomes, and learning satisfaction. Structural equation modeling (SEM) via AMOS was used to construct a full model to explore the relationships between students’ perceptions of the instructor’s role, learning outcomes, and learning satisfaction, as mediated by ASE. As suggested by Schreiber et al. (2006), model fit is acceptable if the values of the comparative fit index (CFI) and the Tucker–Lewis index (TLI) exceed 0.90 and the value of the root mean square error of approximation (RMSEA) is smaller than 0.08. All of the results are explained in the context of the effect size according to Gignac and Szodorai’s (2016) suggested guidelines (small = 0.10 to <0.20, medium = 0.20 to <0.30, large = ≥ 0.30).

## Results

### Validity and Reliability

The 17 items were subjected to EFA with oblimin rotation using the maximum likelihood method. The Kaiser–Meyer–Olkin (KMO) measure verified the sampling adequacy for the analysis: KMO = 0.96, and all KMO values for individual items were higher than.79. Bartlett test of sphericity [χ^2^(136) = 99,473.16, *p* < 0.001] indicated that the factor analysis was appropriate. An initial analysis was run to obtain eigenvalues for each component of the data. Three components had eigenvalues over Kaiser’s criterion of 1, and together they explained 72.73% of the variance.

Based on the factor structure determined by EFA, AMOS 22.0 was used to carry out CFA of the latent variables. A series of CFAs were conducted to test the factor structure of each standardized measure and determine the distinctiveness of the variables in this study. Table 1 presents the results of the reliability and validity tests. All of the measures showed a good fit to the data and had acceptable levels of internal consistency and overall validity.

In terms of reliability, the Cronbach α coefficients of all of the factors ranged from 0.88 to 0.95, and the composite reliability coefficients ranged from 0.91 to 0.93, indicating that all of the constructs exhibited a relatively high level of internal consistency (Fornell and Larcker, 1981).

In terms of validity, the estimates of the square multiple correlation were over the required threshold level of 0.05, indicating that the constructs had acceptable item reliability. The standardized factor loadings of all of the items on their specific constructs, ranging from 0.69 to 0.95, were found to be statistically significant (*p* < 0.001), and all estimates of average variance extracted (AVE), which ranged from 0.62 to 0.87, were higher than the threshold level of 0.50. Therefore, the amount of variance explained by the items of measurement of the variables was greater than the amount of variance produced by measurement error (Fornell and Larcker, 1981), indicating that the constructs had high convergent validity. Discriminant validity was determined by examining whether the square root of the AVE was greater than the intercorrelations of the constructs. As Table 2 demonstrates, the estimates for the square root of the AVE exceeded the correlation coefficients of each pair of constructs in all cases, offering supportive evidence of discriminant validity among the variables.

**TABLE 2:** Descriptive statistics, correlations, and square roots of AVEs of the factors.

	Instructor performance	Instructional support	Instructor innovation	Academic self-efficacy	Perceived learning outcomes	Learning satisfaction
Instructor performance	(0.82)					
Instructional support	0.80**	(0.79)				
Instructor innovation	0.78**	0.73**	(0.87)			
Academic self-efficacy	0.66**	0.71**	0.70**	(0.85)		
Perceived learning outcomes	0.56**	0.68**	0.74**	0.73**	(0.93)	
Learning satisfaction	0.58**	0.73**	0.69**	0.73**	0.76**	(0.86)
Mean	4.13	3.88	3.80	3.45	3.40	3.34
Standard deviation	1.04	1.13	1.03	1.11	1.03	1.16

**p < 0.01. The square roots of AVEs in parentheses along the diagonal.

### Descriptive Statistics and Correlations

Table 2 presents descriptive statistics for and correlations between the variables in this study. Overall, the mean scores for all of the subscales were above the midpoint (3) of a 5-point scale, which indicated that the students gave high scores for their perceptions of all of the factors. Of the three subscale measures of the instructor’s role, instructor performance (mean = 4.13, *SD* = 1.04) had the highest mean score, followed by instructional support (mean = 3.88, *SD* = 1.13) and instructor innovation (mean = 3.80, *SD* = 1.03). Of the students’ perceived learning outcomes, ASE scored the highest (mean = 3.45, *SD* = 1.11), followed by student perceived learning outcomes (mean = 3.40, *SD* = 1.03) and learning satisfaction (mean = 3.34, *SD* = 1.16).

The correlations of the variables, as outlined in Table 2, were below the threshold value of 0.08. The results showed that instructor performance, instructional support, and instructor innovation were positively related to students’ learning outcomes, all with large effect sizes.

### SEM Analysis

**FIGURE 1:**
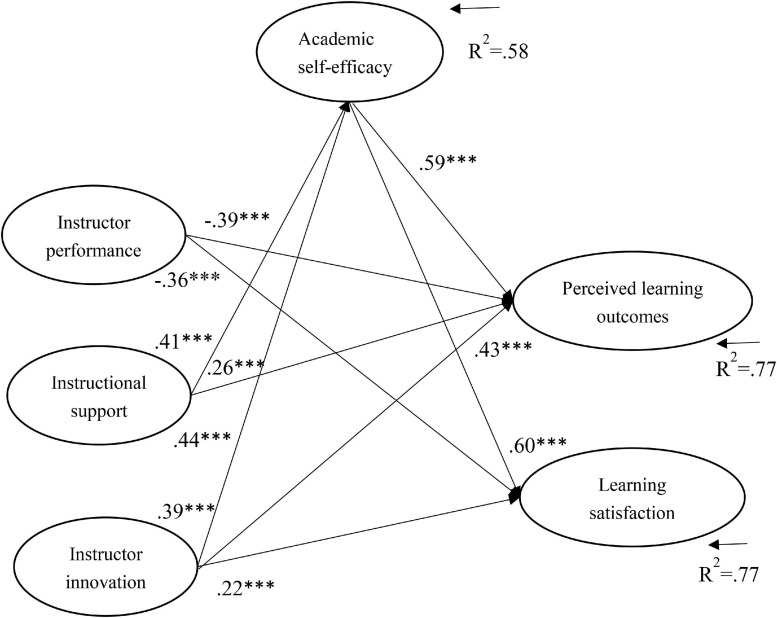
SEM model results showing significant regression paths. *p < 0.05, **p < 0.01, ***p < 0.001; goodness-of-fit indices: χ^2^ = 8081.57, *df* = 262, *p* < 0.001, CFI = 0.95, TLI = 0.94, RMSEA = 0.064.

Structural equation modeling analysis was performed using AMOS 22.0 to examine the relationships between the variables. The model was based on the assumption that correlations were allowed between the independent variables (instructor performance, instructional support, and instructor innovation), the dependent variables (student perceived learning outcomes and learning satisfaction), and the mediator (ASE). The model (Figure 1) provided an acceptable fit to the data (χ^2^ = 17189.93, *df* = 363, *p* < 0.001, CFI = 0.92, TLI = 0.91, RMSEA = 0.080), with the explained variance of 0.77 (students’ perceived learning outcomes), 0.77 (learning satisfaction), and 0.58 (ASE). Figure 1 presents a path diagram showing the relationships between the variables. The SEM results showed that instructor performance was negatively associated with student perceived learning outcomes (β = −0.39, *p* < 0.001) and learning satisfaction (β = −0.36, *p* < 0.001). Instructional support was positively related to self-efficacy (β = 0.41, *p* < 0.001) and students’ perceived learning outcomes (β = 0.26, *p* < 0.001). Instructor innovation was also positively related to ASE (β = 0.39, *p* < 0.001), students’ perceived learning outcomes (β = 0.43, *p* < 0.001), and learning satisfaction (β = 0.22, *p* < 0.001). Self-efficacy was positively related to students’ perceived learning outcomes (β = 0.59, *p* < 0.001) and learning satisfaction (β = 0.60, *p* < 0.001). The effect sizes of these associations were at medium and large levels.

### Mediation Analysis

Mediation analysis based on 5,000 bootstrapping samples was conducted using AMOS 22.0 to estimate the mediation effect of student ASE on the relationships between the instructor’s role and the dependent variables (students’ perceived learning outcomes and learning satisfaction). The effect size of the mediation was measured by the point estimate of the indirect effect. Hayes (2009) suggested that an indirect effect is significant if 0 does not lie between the lower and upper bounds of the 95% confidence interval. The results of this study’s mediation analysis, summarized in Table 3, indicated that students’ ASE significantly mediated the influence of instructional support and instructor innovation on perceived learning outcomes and learning satisfaction. These mediation effects were medium-sized. However, the mediation effect of student ASE on the relationships between instructor performance and students’ perceived learning outcomes and learning satisfaction was not significant.

**TABLE 3:** The estimates of direct effects and indirect effects of the 95% confidence intervals.

Dependent variable	Independent variable	Direct effect	Indirect effect	95% CIs		*R*^2^
				Lower 2.5%	Upper 2.5%	
Perceived learning outcomes	Instructor performance	–0.39	0.01	–0.02	0.05	0.77
	Instructional support	0.26	**0.24**	0.21	0.28	
	Instructor innovation	0.44	**0.23**	0.20	0.26	
Learning satisfaction	Instructor performance	–0.36	0.01	–0.02	0.05	0.77
	Instructional support	0.44	**0.25**	0.22	0.28	
	Instructor innovation	0.22	**0.23**	0.20	0.26	

*Bold items showing significant mediation effect.*

## Discussion

The purpose of this study was to examine the relationships between the perceived role of the instructor and the learning outcomes of university students in cloud-based virtual classrooms in mainland China. The results revealed the positive influence of instructional support and instructor innovation and the negative influence of instructor performance on students’ perceived learning outcomes and learning satisfaction. Student self-efficacy mediated the influence of instructional support and instructor innovation on students’ perceived learning outcomes and learning satisfaction. The results enhance understanding of the role of instructors in student learning outcomes in synchronous learning environments and suggest ways to foster desirable learning outcomes, especially in higher education settings.

### The Relationships Between the Role of Instructors and Students’ Perceived Learning Outcomes and Learning Satisfaction

The observed positive effect of instructional support on students’ perceived learning outcomes is consistent with the findings of previous research on online learning environments (Mullen and Tallent-Runnels, 2006; Martin et al., 2018). Despite wide acceptance of the positive role of instructional support and adequate access to that support, research has indicated that it is particularly important for instructors, especially in online learning environments, to provide timely feedback for and communicate promptly with students to ensure that students receive the proper knowledge and feel supported (Lee et al., 2011; Martin et al., 2019). In synchronous online learning environments, cloud-based technology, such as Rain Classroom (a cloud-based synchronous online learning application commonly used in China, providing bullet-style subtitles), Tecent Docs, and WeChat, has been found to support instructors’ provision of timely feedback and communication, offering developmentally appropriate experiences that enhance students’ behavioral and cognitive engagement (Xu et al., 2020).

This study revealed no significant relationship between the level of instructional support and students’ learning satisfaction. This finding is inconsistent with studies that have demonstrated the positive effect of instructional support on learning satisfaction in online learning settings (Mullen and Tallent-Runnels, 2006; Lee et al., 2011). Research has identified two types of instructional support—academic and affective support—as crucial in developing students’ appropriate learning experiences and promoting their learning, particularly in online settings (Lee et al., 2011). However, a further review of the items measuring instructional support in this study revealed that only instructional academic support was relevant. A possible explanation for our finding is that this single type of support may be suitable only for students who prefer instructor-supported learning experiences (Lee et al., 2011).

The observed positive relationship between students’ perceptions of instructional innovation and their learning satisfaction echoed the findings of Lee (2011). Unlike Lee, however, this study reported an insignificant relationship between instructor innovation and students’ school grades but revealed a positive relationship between students’ perception of instructional innovation and their perceived learning outcomes. Although both students’ perceived learning outcomes and school grades are key criteria used in course evaluation (Kruger and Dunning, 1999), students’ perceptions of learning outcomes can provide valuable insights to inform course development (Rundle-Thiele, 2005). The beneficial effect of instructor innovation does not necessarily translate into an improvement in students’ grades (De Ketele, 2002); however, the results of this study provide evidence that instructor innovation improves students’ learning outcomes in synchronous online settings. Moreover, the results echo the finding of previous research that instructor innovation designed to readapt student learning, improve communication with students, and engage and support students was connected to improved student learning and satisfaction (Walder, 2014). In cloud-based virtual classrooms, therefore, it is crucial for instructors to make good use of cloud computing tools (e.g., Rain Classroom, Tecent Docs, or WeChat) to engage students in meaningful and playful ways to achieve their learning goals. The required innovations in teaching practices resulting from the use of new cloud computing technology are the main barriers to instructors (Al-Samarraie and Saeed, 2018).

This study revealed a significant negative relationship between instructor performance and students’ perceived learning outcomes and learning satisfaction in cloud-based virtual classrooms. Most previous research has revealed a significant positive relationship between instructor performance and students’ learning satisfaction in asynchronous online learning settings, indicating the need for experienced professional instructors to improve students’ learning satisfaction (e.g., Ali and Ahmad, 2011). However, some researchers have argued that the relationship between instructor performance and students’ learning satisfaction may be more complex because of effects arising from their subjective feelings. Rundle-Thiele (2005) indicated that students’ ratings of teaching were lower when they felt that their learning workload and obstacles were greater. Blazar (2018) also suggested that teachers who are skilled in improving students’ achievement may reduce students’ happiness or engagement in class. Therefore, even if instructors’ performance is favorable, students may feel less satisfied if their learning workload increases.

### ASE as a Mediator of the Relationships Between the Instructor’s Role and Students’ Perceived Learning Outcomes and Learning Satisfaction

This study extends knowledge of the effects of students’ ASE from traditional face-to-face classroom learning and asynchronous online learning to cloud-based virtual classrooms. The results revealed that students’ ASE mediated the influence of instructional support and instructor innovation on students’ perceived learning outcomes and learning satisfaction. This indicates that students’ ASE can increase the extent to which students’ perceptions of the instructor’s role explain their online learning outcomes. The results of mediation analysis revealed that the mediation effect of ASE was medium-sized. Thus, the direct effects of instructional support and instructor innovation on students’ perceived learning outcomes and learning satisfaction were significantly actualized by the students’ increased ASE in cloud-based virtual classrooms.

Although very few studies have explored the relationships between these variables in synchronous learning environments, important evidence has been provided that instructional support enhances student ASE in asynchronous online learning environments (Schmidt and Ford, 2006). Synchronous online learning tools facilitating students’ anonymous participation in activities and instructors’ timely feedback, such as bullet subtitles and instant feedback on course content in Rain Classroom, have been widely used in higher education in mainland China since the outbreak of COVID-19. Researchers have suggested that scaffolding learning using cloud computing tools can develop learners’ knowledge construction and mastery experiences (Azevedo et al., 2005; Baanqud et al., 2020), which are among the key sources of self-efficacy. The use of these scaffolding tools supports students’ ASE in online learning by helping them to overcome anxiety and increasing their interaction and engagement with the course material. However, these outcomes are mainly dependent on the online instructional design (Castro and Tumibay, 2019). Therefore, university students’ perceptions of instructional support and instructor innovation are strong predictors of their ASE in online learning, which in turn fosters desirable learning outcomes and learning satisfaction.

The results of this study also revealed a positive relationship between student ASE and learning outcomes and learning satisfaction in synchronous online learning environments. This suggests that university students with greater ASE are more likely to perceive better learning outcomes and be more satisfied with the teaching and learning process. It should be noted that although student ASE has commonly been found to significantly predict academic success in online learning (e.g., Tsai, 2011), a few studies have indicated that ASE does not predict Internet-based learning success (e.g., Yukselturk and Bulut, 2007). However, based on their qualitative analyses of interviews with instructors, Yukselturk and Bulut (2007) maintained that ASE may still affect learning success. Nevertheless, additional work in the field is necessary to reveal the effect of ASE on learning outcomes, particularly in synchronous learning environments.

Despite the large effect sizes of the direct negative effects of perceived instructor performance on students’ perceived learning outcomes and learning satisfaction, the results of the mediation analysis revealed no significant mediating influence of student self-efficacy on those relationships. Similarly, instructor performance was not significantly related to students’ self-efficacy, suggesting a significant direct effect of instructor performance on students’ learning outcomes and satisfaction. As research on the role of teachers in promoting students’ self-efficacy in online learning settings is limited, more research is expected in the near future to explore other potential relationships between the variables, particularly in cloud-based virtual classrooms.

### Limitations of the Study and Directions for Future Research

This study offers several insights into the relationships between students’ perceptions of the role of their instructors and their own learning outcomes in cloud-based virtual classrooms in mainland China. Some limitations, which indicate directions for future research, should be noted. As the design of the study was cross-sectional, a supplemental longitudinal study may be required to confirm the consistent causal relationships between the variables. The results of this study were based on the self-reports of university students, who might have either exaggerated or underreported their perceptions for a number of reasons. Using multiple methods, such as a mixed-methods design, could yield more objective material data. Based on Bandura’s social cognitive theory (1982), the triadic reciprocal causation among environment, behavior, and personal factors suggests that there are various possible models of the interaction effect between the role of the instructor, ASE, and learning outcomes. Accordingly, further investigation of the interaction effects among the three variables is required to gain deeper insights into these interrelationships.

## Conclusion and Implications for Practice

This study investigated the relationships between university students’ perceptions of instructor performance, instructional support, and instructor innovation and their perceived self-efficacy, learning outcomes, and learning satisfaction in cloud-based virtual classrooms. Students’ perceived learning outcomes and learning satisfaction were found to be positively related to instructor innovation but negatively related to instructor performance. Instructional support was positively related to perceived learning outcomes but not directly related to learning satisfaction. Student self-efficacy significantly mediated the effects of instructional support and instructor innovation on students’ perceived learning outcomes and learning satisfaction. The results of this study have several implications for improving university synchronous online learning environments and student learning outcomes.

The positive relationships between university students’ perceptions of instructor innovation and students’ perceived learning outcomes and learning satisfaction highlight the important role played by instructors in facilitating desirable outcomes in online learning environments. Al-Samarraie and Saeed (2018) suggested that the main barrier to instructors in online learning settings is a lack of competence with the relevant technologies. Instructors should be encouraged to take advantage of synchronous online teaching tools during course design, assessment and evaluation, and facilitation to provide more instructional support for students’ learning process. Specific strategies include timely response and feedback, increased instructor availability and presence, timely communication with students, and innovations in online course design and teaching practice. Such practices support collaborative learning by stimulating online discussion and creating a more favorable learning environment with improved learning outcomes that match educational goals (Martin et al., 2019).

The positive relationship between students’ perceived instructional support and learning outcomes indicates that during online course design, assessment, and evaluation, instructors should consider providing multiple forms of support to meet students’ needs and facilitate their online learning. This may enable students to access a learning experience tailored to their learning styles. In addition, the potential detrimental effect of students’ perceptions of instructor performance on students’ perceived learning outcomes and learning satisfaction should not be neglected; it highlights the need to assign an appropriate learning workload and be aware of difficulties faced by students. In addition, course designers should consider students’ needs, and faculty should use a variety of assessment methods, such as rubrics, peer review, and learning analytics, to assess students and ensure that their workload is reasonable (Martin et al., 2019).

Finally, the significant mediation effect of self-efficacy indicates that improving students’ self-efficacy may enhance the beneficial effect of their perceptions of the instructor’s role on online learning outcomes. Evaluations of online teaching and learning could thus incorporate ratings of students’ confidence in the success of their online learning and instructors’ recognition and appreciation of students’ ASE in online learning.

## Data Availability Statement

The raw data supporting the conclusions of this article will be made available by the authors, without undue reservation.

## Ethics Statement

The studies involving human participants were reviewed and approved by the University of Shandong. The participants provided their written informed consent to participate in this study.

## Author Contributions

All the authors contributed to the study design and approved the final version of the manuscript for submission. CL and HX collected the data. JH and RW analyzed the data, drafted and revised the manuscript.

## Conflict of Interest

The authors declare that the research was conducted in the absence of any commercial or financial relationships that could be construed as a potential conflict of interest.
